# Combined Aortic Root Replacement and Heart Transplantation in a Patient with Dilated Cardiomyopathy and Aortic Root Aneurysm

**Published:** 2018-08-01

**Authors:** B. Baharestani, A. Amin, B. Ghadrdoost, M. Behjati

**Affiliations:** 1 Cardiovascular Surgery Department, Rajaie Cardiovascular Medical and Research Center, Iran University of Medical Sciences, Tehran, Iran; 2 Rajaie Cardiovascular Medical and Research Center, Iran University of Medical Sciences, Tehran, Iran; 3 Echocardiography Research Center, Rajaie Cardiovascular Medical and Research Center, Iran University of Medical Sciences, Tehran, Iran

**Keywords:** Heart transplantation, Aortic aneurysm, Aortic root replacement

## Abstract

Concomitant replacement of the ascending aorta and heart transplantation are an infrequent procedure. This procedure was most often performed in patients with Marfan syndrome, however, it has its own technical difficulties. Hereby, we report on combined heart transplantation and aortic root replacement using donor’s ascending aorta in a 25-year-old man diagnosed with dilated cardiomyopathy and ascending aorta aneurysm. This procedure was successful and beneficial to patients with aortopathy who are candidates for heart transplantation.

## INTRODUCTION

Concomitant heart transplantation and ascending aorta replacement are extremely rare [1, 2]. Ascending aorta aneurysm is common in patients with Marfan syndrome. If it is associated with end-stage heart failure, it would be associated with reluctance for placement of patient in cardiac transplantation list [3]. Hereby, we report on a concomitant heart transplantation and aortic root replacement in a patient with dilated cardiomyopathy and ascending aorta aneurysm. 

## CASE PRESENTATION

**Figure 1:**
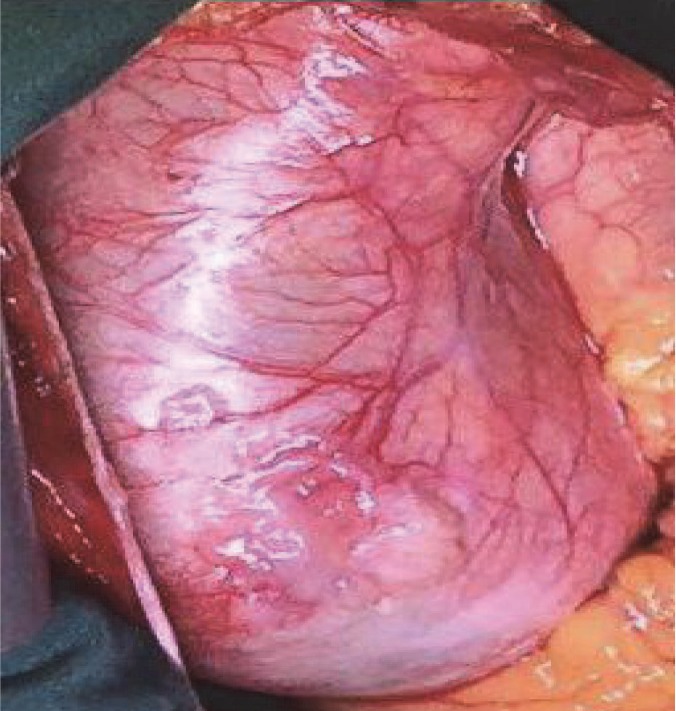
Surgically excised specimen of aneurysmal dilatation of the ascending aorta

A 25-year-old man, known case of dilated cardiomyopathy, aneurysm of the ascending aorta, and severe aortic regurgitation, referred to our center for heart transplantation. He had no risk factor for cardiovascular diseases. He was under treatment with furosemide, spironolactone, lisinopril, atorvastatin, and carvedilol. Echocardiography showed severe left ventricular enlargement with severe systolic dysfunction (LVEF of 20%), global hypokinesia, significantly increased left ventricular filling pressure, severe right ventricular enlargement with severe systolic dysfunction, tethered mitral leaflets with moderate functional mitral regurgitation, tricuspid malcoapted aortic leaflets with severe aortic insufficiency, aneurysmal dilatation of the sinus of Valsalva (6.9 cm) and ascending aorta (8.1 cm), severe pulmonary hypertension (mean PAP of 45 mm Hg), and large bilateral pleural effusion. He underwent total cardiac and aortic root transplantation. The surgically excised aortic aneurysm is shown in Figure 1. 

Operative Technique

After proper prep and drape, midline sternotomy was done with 3 cm extension of superior aspect of the incision to the neck. Using two thoracic retractors, at the same time, we had a good exposure to the thorax and neck vessels. Before opening the pericardium, we explored the innominate artery in the neck and after injection of the proper amount of heparin, we cannulated it directly with simple aortic cannula. At first, tip of the cannula was guided toward the aortic arch. Then, the pericardium was opened vertically and a two-stage cannula was inserted into the right atrium. Cardiopulmonary bypass was started and the patient cooled down to 28 °C.

The groove between the ascending aorta and pulmonary artery was dissected properly and an aortic clamp was inserted at the mid-portion of the most dilated part of the ascending aorta. Resection of the recipient’s heart was done as routine with the resection of aorta just below the clamp. The donor’s heart was transplanted first with the left atrium anastomosis. Then, the posterior part of the pulmonary artery was anastomosed properly. Then, the tip of the aortic cannula was turned toward the cervical vessels at low flow state and aortic clamp was opened. Another clamp was inserted between the arch and the cannula at innominate artery. Circulatory arrest was started with a perfusion rate of 600 mL/min of head and neck vessels. Hemi-arch resection was done and the donor’s aorta, which was resected totally with its arch, was anastomosed to the hemi-arch of the patient.

Warming of the patient was started. The cannula’s tip was rotated again toward the aortic arch. Normal cardiopulmonary flow was started after inserting the aortic vent and the superior and inferior vena cava and anterior part of the pulmonary artery was anastomosed as routine at normal temperature. The cardiopulmonary bypass was stopped. Immediately post-operative trans-esophageal echocardiography showed normal left ventricular size with mild to moderate systolic dysfunction (LVEF of 40%–45%) with hypokinesia of the lateral wall, moderate right ventricular enlargement with moderate to severe systolic dysfunction, mild to moderate mitral regurgitation, no aortic insufficiency, and normal ascending aorta. The patient had an uneventful hospital course. He was followed for three years annually until now. His last admission was for annual cardiac biopsy, when echocardiography showed normal left ventricular size and systolic function (LVEF of 55%), mild to moderate right ventricular enlargement and dysfunction, no aortic insufficiency, mild mitral regurgitation, and no pulmonary artery hypertension. 

## Discussion

There are only a few reports of combined heart and aortic replacements globally in patients with diagnosis other than Marfan syndrome. Successful procedure needs a through harvest of donor’s heart and ascending aorta, which bypasses the need for insertion of prosthetic conduit graft. This technique is much simpler and more cost-benefit. This procedure would diminish the reluctance for placing the patient with aortopathy in transplantation waiting list. 
